# Targeted Deletion of Vesicular GABA Transporter from Retinal Horizontal Cells Eliminates Feedback Modulation of Photoreceptor Calcium Channels^1^,^2^,^3^

**DOI:** 10.1523/ENEURO.0148-15.2016

**Published:** 2016-03-10

**Authors:** Arlene A. Hirano, Xue Liu, Jim Boulter, James Grove, Luis Pérez de Sevilla Müller, Steven Barnes, Nicholas C. Brecha

**Affiliations:** 1 Department of Neurobiology, David Geffen School of Medicine, University of California, Los Angeles, Los Angeles, California 90095; 2 Veterans Administration Greater Los Angeles Healthcare System, Los Angeles, California 90073; 3 Department of Psychiatry and Biobehavioral Sciences, Hatos Research Center for Neuropharmacology, Semel Institute for Neuroscience and Human Behavior, David Geffen School of Medicine, University of California, Los Angeles, Los Angeles, California 90095; 4 Department of Physiology and Biophysics, Dalhousie University, Halifax, Nova Scotia B3H 4R2, Canada; 5 Department of Ophthalmology & Visual Sciences, Dalhousie University, Halifax, Nova Scotia B3H 4R2, Canada; 6 Jules Stein Eye Institute, David Geffen School of Medicine, University of California, Los Angeles, Los Angeles, California 90095; 7 Department of Medicine, David Geffen School of Medicine, University of California, Los Angeles, Los Angeles, California 90095

**Keywords:** Ca channels, Cx57-iCre, GABA receptors, inhibitory feedback, retinal horizontal cells, synaptic vesicles

## Abstract

The cellular mechanisms underlying feedback signaling from horizontal cells to photoreceptors, which are important for the formation of receptive field surrounds of early visual neurons, remain unsettled. Mammalian horizontal cells express a complement of synaptic proteins that are necessary and sufficient for calcium-dependent exocytosis of inhibitory neurotransmitters at their contacts with photoreceptor terminals, suggesting that they are capable of releasing GABA via vesicular release. To test whether horizontal cell vesicular release is involved in feedback signaling, we perturbed inhibitory neurotransmission in these cells by targeted deletion of the vesicular GABA transporter (VGAT), the protein responsible for the uptake of inhibitory transmitter by synaptic vesicles. To manipulate horizontal cells selectively, an iCre mouse line with Cre recombinase expression controlled by connexin57 (Cx57) regulatory elements was generated. In *Cx57-iCre* mouse retina, only horizontal cells expressed Cre protein, and its expression occurred in all retinal regions. After crossing with a *VGAT^flox/flox^* mouse line, VGAT was selectively eliminated from horizontal cells, which was confirmed immunohistochemically. Voltage-gated ion channel currents in horizontal cells of *Cx57-VGAT^−/−^* mice were the same as *Cx57-VGAT^+/+^* controls, as were the cell responses to the ionotropic glutamate receptor agonist kainate, but the response to the GABA_A_ receptor agonist muscimol in *Cx57-VGAT^−/−^* mice was larger. In contrast, the feedback inhibition of photoreceptor calcium channels, which in control animals is induced by horizontal cell depolarization, was completely absent in *Cx57-VGAT^−/−^* mice. The results suggest that vesicular release of GABA from horizontal cells is required for feedback inhibition of photoreceptors.

## Significance Statement

Feedback inhibition by horizontal cells regulates the photoreceptor calcium channels responsible for the release of the neurotransmitter glutamate. This feedback inhibition contributes to the formation of the receptive field surrounds of early visual neurons, which is important for contrast sensitivity and color opponency in vision. The cellular mechanisms underlying feedback inhibition are not fully understood. One disputed mechanism for mammalian horizontal cell inhibitory output involves the vesicular release of the inhibitory neurotransmitter GABA. By selectively deleting the transporter that normally loads GABA into vesicles in horizontal cells, we show that horizontal cells lose their ability to modulate photoreceptor calcium signals. These findings implicate a role for the vesicular release of GABA in mediating inhibitory feedback.

## Introduction

Output from photoreceptors occurs at a synaptic complex composed of the photoreceptor synaptic terminal, bipolar cell dendrites, and horizontal cell endings, forming the initial site of visual information transfer and processing. The ON bipolar dendrites and horizontal cell endings invaginate the photoreceptor terminal. Horizontal cells are known to feed inhibition back onto photoreceptors, but the cellular mechanisms of feedback inhibition in mammals are not fully understood (Thoreson and Mangel, 2012; Liu et al., 2013). We do know that horizontal cell inhibition modulates the presynaptic voltage-gated calcium channels in photoreceptors, which control the release of glutamate from those cells (Thoreson and Mangel, 2012). Evidence exists for several feedback mechanisms at this synapse, including an ephaptic effect and a HEPES-blocked, pH-sensitive mechanism, but a feedback pathway involving vesicular release of an inhibitory neurotransmitter, such as GABA, remains controversial (Thoreson and Mangel, 2012; Liu et al., 2013; Wang et al., 2014; Kemmler et al., 2014).

To address the role of GABA release from mammalian horizontal cells, we created a mouse line in which the vesicular GABA transporter (VGAT) was deleted in horizontal cells, thus preventing them from loading synaptic vesicles with GABA and, hence, knocking out their ability to release GABA by a regulated vesicular mechanism. To do this, we took advantage of the selective expression of the connexin57 (*Cx57*) gene in retinal horizontal cells (Hombach et al., 2004; Ciolofan et al., 2007) by exchanging the protein-coding region of the *Cx57* gene with the “improved” Cre recombinase (*iCre*) gene (Shimshek et al., 2002), such that Cre expression occurred specifically in horizontal cells. Crosses with floxed VGAT mice (Tong et al., 2008) generate mice that have horizontal cells in which VGAT is deleted.

Evidence for GABA receptors and direct actions of GABA on mammalian photoreceptors has been negative or at best equivocal (for review, see Wässle et al., 1998; Thoreson and Mangel, 2012; Kemmler et al., 2014). Renewed motivation to determine whether there is a role for GABA release in feedback inhibition from mammalian horizontal cells comes in part from numerous confirmatory observations in nonmammalian vertebrate retinae (Thoreson and Mangel, 2012), as well as a preponderance of evidence showing that the cellular proteins typically responsible for Ca^2+^-regulated vesicular release of GABA are present in horizontal cells, including SNAP-25; syntaxin-1a; syntaxin-4; VAMP-1 (vesicle-associated membrane protein 1); GAD; VGAT; and L-, N- and P/Q-type Ca^2+^ channels in mammalian horizontal cells (Haverkamp et al., 2000; Cueva et al., 2002; Jellali et al., 2002; Johnson et al., 2003; Hirano et al., 2005; 2007; 2011; Sherry et al., 2006; Guo et al., 2009; 2010; Lee and Brecha, 2010; Liu et al., 2013).

In mammalian retina, there is evidence of a mechanism in which GABA release and autoreception signaling by horizontal cells can lead to changes in the pH of the synaptic cleft. Ionotropic GABA_A_ receptors (GABA_A_Rs) and actions have been demonstrated in mouse and rat horizontal cells (Feigenspan and Weiler, 2004; Liu et al., 2013), and it has been proposed that GABA has an indirect role in producing feedback inhibition of photoreceptor Ca channels (Liu et al., 2013). Depolarization of horizontal cells with the ionotropic glutamate receptor (iGluR) agonist kainate activated their voltage-gated Ca channels, and inhibited photoreceptor calcium signals in rat retinal slices in a HEPES-sensitive manner. Antagonists of GABA_A_ receptors blocked this action. The intermediary mechanism of changing cleft pH is hypothesized to be due to the fact that GABA_A_ receptors possess relatively high permeability for bicarbonate (HCO_3_^−^), as well as chloride (Cl^−^; Bormann et al., 1987), and the resulting flux of HCO_3_^−^ across the horizontal cell membrane changes the pH in the synaptic cleft between the horizontal cell and photoreceptor axon terminal. Even small changes of pH in the cleft modulate the gating of photoreceptor Ca channels, leading to altered glutamate release from these cells (Barnes et al., 1993). Superfusion with HEPES buffer clamps the extracellular pH in the cleft, and the prevention of pH changes blocks inhibitory feedback to photoreceptors (Vessey et al., 2005; Thoreson and Mangel, 2012).

Here we show that the Cre recombinase protein is selectively expressed in horizontal cells of *Cx57-iCre* mice and, following crosses of *Cx57-iCre* mice with a *VGAT^flox/flox^* mouse line, VGAT immunoreactivity is absent from the horizontal cells and their processes in the outer plexiform layer (OPL); and that the intrinsic electrophysiological properties of the *Cx57-VGAT^−/−^* horizontal cells are normal. Testing the role of horizontal cell-released GABA in photoreceptor calcium channel modulation, we show that the loss of VGAT from horizontal cells eliminates the inhibitory feedback of photoreceptor calcium channels.

## Materials and Methods

### Animal use statement

Electrophysiological, imaging and immunohistochemical experiments were performed in accordance with the guidelines for the welfare of experimental animals issued by the U.S. Public Health Service Policy on Human Care and Use of Laboratory Animals (2002), the University of California, Los Angeles Chancellor’s Animal Research Committee, and the Canadian Council on Animal Care.

### Generation of the targeting vector

The genomic DNA clone of the mouse connexin 57 (*Cx57, Gja10*) gene was isolated from adult 129/SvJae mouse liver genomic DNA. A Sau3AI restriction endonuclease fragments encoding the *Gja10* gene (GenBank accession #NM_010289) was obtained from a mouse strain 129S4/SvJae genomic DNA library (Stratagene) and subcloned into the vector λ FIX II (catalog #248211, Stratagene). Three genomic clones (MG801, MG806, MG811) containing the Cx57 coding sequence (CDS) were sequenced to determine the physical map. A 8133 bp NheI restriction endonuclease fragment of MG801 (15,946 bp insert) was subcloned into pBS SK[−] (Stratagene) to produce the pCx57.1 construct.

The protein-coding region of the Cx57 gene was replaced precisely by NcoI and NdeI restriction endonuclease digestion with the improved Cre recombinase gene (where the codon usage has been optimized for expression in mammalian cells; Shimshek et al., 2002). The *iCre* gene was obtained from pBOB-CAG-iCRE-SD (plasmid ID no. 12336; Addgene). Finally, a positive selection phosphoglycerate kinase (PGK) promoter–neomycin (neo) resistance cassette flanked by two Flp recombinase recognition (FRT) sites was inserted upstream of the iCre gene. The 2FRT-PGK neo cassette was obtained from ploxP-2FRT-PGKneo (originally a gift from S. Fiering, Dartmouth College, Hanover, NH). This construct was subcloned into the targeting vector pKO-Select DT (Lexicon Genetics). This targeting construct pCx57.6 was electroporated into 129S4/SvJae embryonic stem (ES) cells, and homologous recombinants were obtained after gentamicin (G418) selection and Southern blot hybridization analyses. The successfully targeted ES cell clones were injected into mouse blastocysts (embryonic day 3.5), which were then implanted into the uterine horns of pseudopregnant female mice. The resultant chimeric male pups were backcrossed to C57BL/6J female mice, and the progeny were scored for germline transmission of the targeted allele by agouti coat color and genotyping for the iCre transgene. The neo selection cassette was excised by crossing with a FLP1 recombinase mouse (stock #009086, The Jackson Laboratory; Farley et al., 2000). The Cx57-iCre mice were backcrossed to C57BL/6J mice (males and females; stock #000664, The Jackson Laboratory).

Routine genotyping of *Cx57-iCre* mice was performed by using tail biopsy tissue DNA samples (DNeasy Tissue Kit; Qiagen), primers Cx57.11 (5'-AGG AAA GTC TCC AAC CTG CTG ACT-3') and Cx57.12 (5'-GCC AAT GTG GAT CAG CATTCT CCC-3'), and HotStarTaq DNA Polymerase (Qiagen) as described by the manufacturer. PCR cycle parameters were as follows: 95°C for 15 min, 55°C for 1 min, and 72°C for 2 min for 1 cycle; 95°C for 0.5 min, 55°C for 1 min, and 72°C for 2 min for 33 cycles; and 95°C for 0.5 min, 55°C for 2.5 min, and 72°C for 5 min for 1 cycle, for a total of 35 cycles. Reaction products were electrophoresed on a 1.5% agarose/TAE gel, stained with ethidium bromide or GelRed (Biotium) and imaged. The PCR fragment length for the *iCre* transgene was 600 bp.

### Mouse lines

*Cx57-iCre* mice were crossed with the Cre reporter lines *R26-mT/-mG*, *Ai9(RCL-tdT)*, or *Ai14/RCL-tdT)-D* (https://www.jax.org/strain/007576, https://www.jax.org/strain/007909, and https://www.jax.org/strain/007914, respectively; The Jackson Laboratory), and *VGAT^flox/flox^* (gift of Dr. Bradford B. Lowell, Beth Israel Deaconess Medical Center, Harvard Medical School; https://www.jax.org/strain/012897). Hemizygous *Cx57-iCre^+/−^:: tdTomato^+/−^* (*Cx57-tdTomato*, which are VGAT^+/+^), *Cx57-iCre^+/−^:: VGAT^+/+^ (Cx57-VGAT^+/+^)*, and *Cx57-iCre^+/−^:: VGAT^flox/flox^* (*Cx57-VGAT^−/−^*) mice were used for this study.

### Immunohistochemical labeling

Adult mice of either sex were used for these studies. Following deep anesthesia with 1-3% isoflurane (IsoFlo; Abbott Laboratories), the eyes were enucleated, and the anterior chamber and lens were removed. The eyecups were immersion fixed in 4% (w/v) paraformaldehyde (PFA) or 2% PLP (2% PFA, 75 mm l-lysine, and 10 mm Na periodate) in 0.1 m phosphate buffer (PB), pH 7.4, for 15-30 min, cryoprotected in 30% sucrose and sectioned vertically at 12-14 μm on a cryostat onto gelatin-coated slides. Immunostaining was performed using retinal sections incubated in a blocking solution containing 10% normal goat serum (NGS), 1% bovine serum albumin (BSA), 0.5% Triton X-100, 0.05% sodium azide (NaN_3_) in 0.1 m PB, pH 7.4 for 1 h. The primary antibody was diluted in 3% NGS, 1% BSA, 0.5% Triton X-100, and 0.05% NaN_3_, in 0.1 m PB, for 12–16 h at room temperature. The specific immunolabeling was visualized using Alexa Fluor 488-, 568- or 594-conjugated anti-rabbit or mouse secondary antibodies (Invitrogen) at 1:500 dilutions for 40-60 min at room temperature. The immunostaining was examined on a Zeiss Axioplan2 fluorescence microscope (Carl Zeiss) with a 25× 0.8 numerical aperture (NA) Plan-NEOFLUAR objective and a Zeiss Laser Scanning Microscope 510 Meta or 710 confocal microscope equipped with krypton/argon and helium/neon lasers with a 40x C-Apochromat/1.2 NA, C-Apochromat 63×/1.2 NA corrected water or Plan-Neofluar 63×/1.25 NA oil objective. Confocal images were acquired with and converted into TIFF files using the Zeiss LSM 510 software. Images were prepared in Photoshop CS4 (Adobe Systems). Antibodies used include a mouse monoclonal against Cre recombinase (1:1000; MAB3120, Millipore) and against calbindin (1:3000; clone CB-955; C9848, Sigma-Aldrich), as well as a rabbit polyclonal antibody against calbindin (1:10,000; CB38A; Swant).

### Patch-clamp recording from isolated horizontal cells

Retinae from td-Tomato-labeled *Cx57- iCre^+/-^::VGAT^+/+^* and *Cx57-iCre^+/-^::VGAT^−/−^* mice were enzymatically and mechanically dissociated. I_K_ and I_Ca_ were measured in identified horizontal cells, using standard patch-clamp protocols (Axopatch 200B, pCLAMP version 8.2). Isolated horizontal cells were prepared following incubation of retinae in HBSS (HyClone) containing 18 U/ml papain and 100 U/ml DNase I (Worthington Biochemical) at 37°C for 40 min. Isolated cells were obtained by gentle trituration after digestion. The cells were kept in DMEM (Life Technologies) with 10% fetal bovine serum (Life Technologies) in a CO_2_ incubator at 37°C. To record voltage-gated K^+^ currents, isolated horizontal cells were bathed with a solution containing the following (in mm): 125 NaCl, 3 KCl, 2 CaCl_2_, 1.25 NaH_2_PO_4_, 1 MgCl_2_, 25 NaHCO_3_, and 10 glucose bubbled continuously with 95% O_2_, 5% CO_2_. The patch pipettes contained the following (in mm): 140 KCl, 0.1 CaCl_2_, 1 EGTA, 10 HEPES, 3 Mg-ATP, 0.2 Li-GTP, and 8 phosphocreatine, at pH 7.2. For recordings of voltage-gated Ca^2+^ channel currents, the bath solution was changed to one that had 10 mm BaCl_2_ added with no added CaCl_2_, and the patch pipettes contained the following (in mm): 140 CsCl, 0.1 CaCl_2_, 1 EGTA, 10 HEPES, 3 Mg-ATP, 0.2 Li-GTP, and 8 phosphocreatine, at pH 7.2, adjusted with CsOH. For recordings of muscimol- and kainate-activated currents, the pipette contained the following (in mm): 140 CsCl, 0.1 CaCl_2_, 1 EGTA, 10 HEPES, and 8 phosphocreatine, at pH 7.2. Room-temperature (21–24°C) solutions were superfused via a gravity-driven fast-flow system. Patch electrodes with 5–10 MΩ tip resistance were pulled from borosilicate glass capillary tubes (A-M Systems) using a micropipette puller (Sutter Instrument). The bath reference electrode consisted of an AgCl wire in a side chamber. Cell voltage was clamped with an Axopatch 200B amplifier (Axon Instruments) using whole-cell capacitance and series resistance compensation. The current signal was filtered at 2 kHz and digitized at 10 kHz with an Axon Digidata 1320A for storage on the hard disk of a computer running pCLAMP version 8.2 acquisition software. Linear leak subtraction was applied to the voltage-gated ion channel recordings at the time of recording or digitally *post hoc* except when analyzing the conductance changes in response to muscimol and kainate.

### Ca^2+^ imaging in retinal slices

Young adult mice (1–2 months) of either sex were deeply anesthetized with 1–3% isoflurane (IsoFlo), the eyes were enucleated, and the anterior portion of an eye, including the lens, was removed. The resulting eyecup was trimmed and a section of retina with scleral backing was placed vitreal side down on a piece of filter (2 × 5 mm, type GS, 0.2 μm pores; Millipore). After the retina had adhered to the filter, the sclera was peeled away, and the retina and filter paper were cut into 150–200 μm slices using a tissue chopper (Tissue Slicer; Stoelting Co.) mounted with a razor blade (No. 121-6; Ted Pella), and the slices were rotated 90° to permit viewing of the retinal layers. Intracellular Ca^2+^ changes were assessed with the Ca^2+^-sensitive dye fluo-4 (Invitrogen), which was prepared as a 1 mm stock solution in DMSO and diluted in mammalian superfusate to a final concentration of 10 μm. Retinal slices were incubated in fluo-4 for 1 h at room temperature in darkness.

Calcium imaging with fluo-4 was performed on retinal slices from *Cx57-iCre^+/−^: VGAT^−/−^* and *Cx57-iCre^+/−^: VGAT^+/+^* mice using a Zeiss LSM5 Pascal system with an IR-Achroplan 40×/0.8 NA water-immersion objective. The 488 nm laser line of the argon laser provided excitation, and the emission was collected through a 505 nm long-pass filter on a photomultiplier tube. Slices were superfused via a gravity-driven system (ALA Scientific) with a solution containing the following (in mm): 125 NaCl, 3 KCl, 2 CaCl_2_, 1.25 NaH_2_PO_4_, 1 MgCl_2_, 25 NaHCO_3_, and 10 glucose bubbled continuously with 95% O_2_, 5% CO_2_. To test the changes of activity of voltage-dependent Ca^2+^ channels in photoreceptors, twin pulses (30 s) of 30 mm K^+^ were superfused in the absence and presence of 50 μm kainate (Sigma-Aldrich) or 50 μm 2,3-dihydroxy-6-nitro-7-sulfonyl-benzo[*f*]quinoxaline (NBQX; Tocris Bioscience). NaCl was reduced by 27 mm to maintain osmolarity. The dose–response relation for the effect of [K^+^]_o_ on photoreceptor [Ca^2+^]_i_ was sigmoidal with an EC_50_ of 17.5 mm K^+^, and the value of 30 mm K^+^ was chosen as it produced large but nonsaturating responses. Images were acquired at 5 s intervals. For analysis, regions of interest (ROIs) were drawn over the photoreceptor somata, and changes in fluorescence were recorded. The change produced in the presence of the test drug during the second of the paired high-K pulses was compared with the change produced during the first high-K pulse and is shown normalized to the first K pulse in figure 5. The common, minor variations of the width of the [Ca^2+^]_i_ response occurred randomly and were not associated with the drug test. Typically, 5–20 ROIs were analyzed in each slice, and all experiments were performed on three to five preparations from separate animals.

### Data analysis

All data are reported as the mean ± SEM, unless otherwise stated. Graphing and statistical analyses were performed using Matlab version 7.6 (MathWorks). Statistical tests were used to determine whether significant differences existed between datasets (specified in Results; Table 1). Normality was assessed quantitatively with a Shapiro–Wilk test and was qualitatively verified with Q-Q plots. *p* values <0.05 were considered to be statistically significant.

**Table 1:** Statistical tests

	Measurement/group	Data structure	Type of test	Confidence interval
a	Kir	Normal	Welch’s *t* test	−257 to 389 (pA)
b	Kv	Normal	Welch’s *t* test	−1822 to 2328 (pA)
c	Cav	Normal	Welch’s *t* test	−139 to 97 (pA)
d	*V*_½_	Normal	Welch’s *t* test	−14.6 to 22.8 (mV)
e	Muscimol WT	Unknown	Wilcoxon signed rank test	1.35–0.14 (nS)
f	Muscimol KO	Normal	Paired *t* test	4.58–0.85 (nS)
g	Muscimol WT vs KO	Normal	Welch’s *t* test	0.57–3.60 (nS)
h	Kainate WT	Normal	Paired *t* test	2.19–0.78 (nS)
i	Kainate KO	Normal	Paired *t* test	2.39–0.21 (nS)
j	Kainate WT vs KO	Normal	Welch’s *t* test	−1.28 to 0.91 (nA)
k	[Ca]_i_ kainate WT	Normal	Welch’s *t* test	−28.1 to −13.5 (%)
l	[Ca]_i_ NBQX WT	Normal	Welch’s *t* test	8.0–44.6 (%)
m	[Ca]_i_ kainate KO	Unknown	Wilcoxon rank sum test	−8.2 to 24.0 (%)
n	[Ca]_i_ NBQX KO	Normal	Welch’s *t* test	−9.3 to 12.8 (%)

## Results

### Generation of the conditional knockout of VGAT in mouse horizontal cells

**Figure 1.**
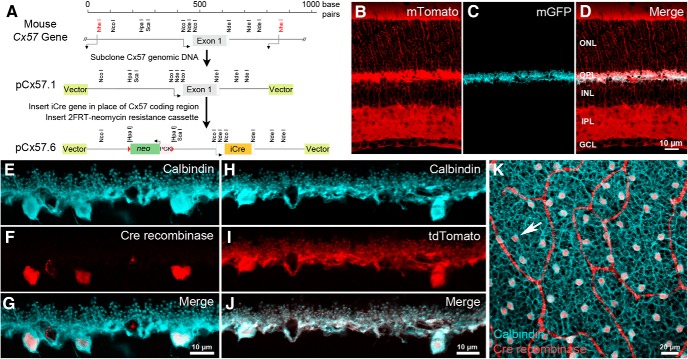
Genetic engineering of *Cx57-iCre* knock-in transgenic mice. ***A***, The Cx57 coding region was removed and replaced with an in-frame, codon-optimized, improved *iCre* gene to produce the mouse Cx57-iCre targeting construct pCx57.6. ***B–D***, In *Cx57-iCre:: ACTB-mT/mG* retinae, non-iCre-expressing cells express membrane-bound tdTomato (mTomato; ***B***), the expression of membrane-bound eGFP (mGFP) appears only in horizontal cells (***C***) within the retina, suggesting that recombination occurred specifically in horizontal cells within the retina (***D***, merge). Note that the overall morphology of the retina appeared normal. ***E–G***, Calbindin immunoreactivity identified horizontal cells (***E***, blue), and Cre immunolabeling (***F***, red) occurred exclusively in horizontal cell bodies (***G***, merge). Scale bar, 10 µm. ***H–J***, *Cx57-iCre* crossed with a Cre-dependent reporter line (*Ai14*, *R26R-tdTomato*) produced tdTomato expression (***I***, red) in horizontal cells (***H***) overlapping perfectly with calbindin immunolabeling (***J***, white). Scale bar, 10 µm. ***K***, Cre expression occurred in horizontal cells in all regions of the retina. Confocal micrograph of a whole-mount retina from a homozygous *Cx57-iCre* mouse labeled with antibodies to calbindin (blue) and Cre recombinase (red). Arrow points to a horizontal cell body containing Cre. Red looping bands are nonspecific labeling of blood vessels by the 2° antibody. Projection of four confocal images; *z*-steps = 0.5 µm. Scale bar, 20 µm. ONL, outer nuclear layer; OPL, outer plexiform layer; INL, inner nuclear layer; IPL, inner plexiform layer; GCL, ganglion cell layer.

Due to the structural complexity of the first synapse in the visual system (Haverkamp et al., 2000; Wässle, 2004) as well as the relatively low cell density of horizontal cells across the mammalian retina (Jeon et al., 1998), it has been difficult to manipulate horizontal cells to investigate the molecular mechanisms underlying their neurotransmission. In the mammalian retina, connexin57, a gap junction protein, is expressed solely in horizontal cells (Hombach et al., 2004). We exchanged the coding region of the Cx57 gene (*Gja10*) with the improved *Cre* recombinase gene (*iCre*) by homologous recombination to generate the *Cx57-iCre* mouse (Fig. 1*A*). Consistent with endogenous Cx57 expression, Cre recombinase protein is selectively expressed in horizontal cells of *Cx57-iCre* mice, as demonstrated in vertical sections of the *Cx57-iCre* retina by the double immunolabeling of *Cx57-iCre* mouse retina with antibodies to Cre and calbindin, a marker for horizontal cells (Fig. 1*E–G*; Röhrenbeck et al., 1987; Haverkamp and Wässle, 2000; Hirano et al., 2011). The calbindin immunolabeling fills the entirety of the horizontal cell, including the soma, processes and the endings that insert into the photoreceptor triad synapse. Cre immunoreactivity is observed within the cell body, as predicted, because of a nuclear localization signal; further, the Cre-immunoreactive cells were present in all areas of the retina (Fig. 1*K*). To demonstrate that the iCre protein was capable of mediating recombination, the *Cx57-iCre* line was crossed with the Cre-reporter line (*R26R-mT/mG*, The Jackson Laboratory; https://www.jax.org/strain/007576), where cells express membrane-bound tdTomato fluorescent protein, except in Cre-expressing cells. In these cells, the tdTomato gene is deleted by the Cre recombinase and the membrane-bound enhanced green fluorescent protein (eGFP) is expressed instead (Fig. 1*B–D*). In the *Cx57-iCre::R26R-mT/mG* retina, the eGFP is confined to the plasma membrane of horizontal cells in the OPL (Fig. 1*C*). When Cre-reporter lines, such as *Ai9(RCL-tdT)* (The Jackson Laboratory; https://www.jax.org/strain/007909) or *Ai14(RCL-tdT)-D* (The Jackson Laboratory; https://www.jax.org/strain/007914; Madisen et al., 2010) are used, strong fluorescent protein expression is observed in horizontal cells. In the *Cx57-iCre::Ai14* retina, tdTomato reporter expression was observed in horizontal cells (Fig. 1*H–J*). Light microscopic analyses of the retinae from these crosses indicated that Cre successfully mediated recombination in horizontal cells, and that the overall retinal morphology was maintained.

### VGAT is selectively eliminated from horizontal cells in the retina

**Figure 2.**
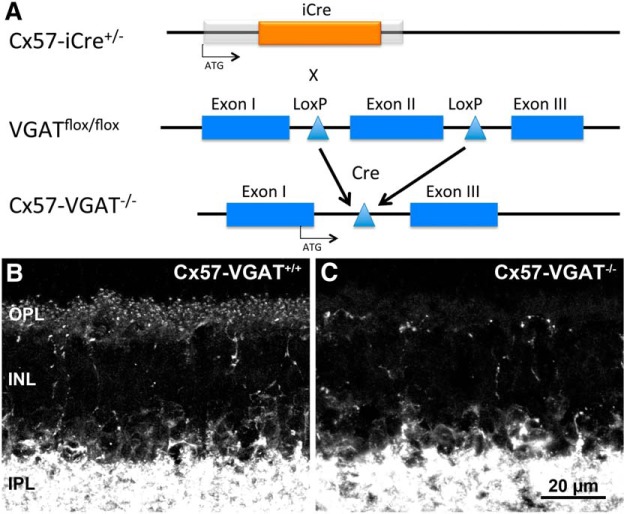
Targeted knockout of VGAT from horizontal cells in *Cx57-VGAT^−/−^* retinae. ***A***, Crosses of *Cx57-iCre^+/-^* and *VGAT^flox/flox^* mouse lines resulted in a conditional knockout of VGAT in horizontal cells in *Cx57-VGAT^−/−^* retinae. ***B***, Vertical section of a *Cx57-VGAT^+/+^* mouse immunolabeled for VGAT in the OPL and IPL. ***C***, VGAT immunolabeling in *Cx57-VGAT^−/−^* retinae is missing from horizontal cell tips, but remains unchanged in the IPL. The remaining VGAT-immunoreactive fibers and puncta in the OPL likely correspond to processes of GABAergic interplexiform cells. Note that both ***B*** and ***C*** show these labeled fibers and puncta. The overall morphology of the retina is unchanged. INL, Inner nuclear layer. Projection of 12 optical sections, *z*-steps = 0.5 µm. Scale bar, 20 µm.

To test whether vesicular GABA release from horizontal cells participates in inhibitory feedback to photoreceptors, we conditionally eliminated VGAT expression in horizontal cells. The *Cx57-iCre* mouse line was crossed with a *VGAT^flox/flox^*mouse line (The Jackson Laboratory; https://www.jax.org/strain/012897), in which exon 2 of the *VGAT* gene is flanked by *loxP* sites and is excised upon recombination (Fig. 2*A*; Tong et al., 2008). Immunolabeling for VGAT in the retinae of *Cx57-iCre* ^+/-^*::VGAT^+/+^* (*Cx57-VGAT^+/+^*) and *Cx57-iCre* ^+/-^*::VGAT^flox/flox^* (*Cx57-VGAT^−/−^*) mice, shown in Figure 2, *B* and *C*, respectively, revealed that VGAT immunoreactivity was absent from the horizontal cells and their processes in the OPL in the *Cx57-VGAT^−/−^* retina. Whereas in both, the amacrine cells in the inner retina and their processes in the inner plexiform layer maintained high levels of VGAT immunoreactivity. Some VGAT-specific staining remained in the OPL of *Cx57-VGAT^−/−^* mice, which is attributable to processes of GABA-releasing interplexiform cells (Witkovsky et al., 2008; Dedek et al., 2009). These processes can be discerned in the control animals as well (Fig. 2*B*,*C*). There was no obvious alteration in the overall morphology and thickness of the synaptic and cellular layers of the retina.

### Electrophysiological properties of *VGAT^−/−^* horizontal cells are unchanged from those recorded in *VGAT^+/+^* mice

**Figure 3.**
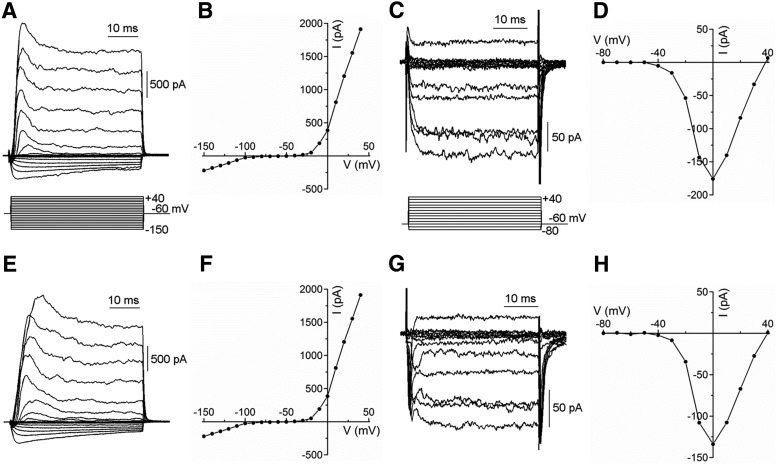
Voltage-gated K^+^ and Ca^2+^ channel currents remain normal in horizontal cells from *Cx57-VGAT^−/−^* retinae. ***A–H***, Whole-cell voltage-clamp recordings from isolated horizontal cells from *Cx57-tdTomato VGAT^+/+^* (***A–D***) and *Cx57-VGAT^−/−^* (***E–H***) mice show that the membrane currents of horizontal cells are unaffected by the deletion of VGAT. Examples of inward and outward K^+^ currents from normal (***A***) and VGAT knock-out (***E***) horizontal cells are similar, and their peak current–voltage (*I–V*) relations (***B***, ***F***) are comparable. Ca channel currents recorded with 10 mm Ba^2+^ in wild-type (***C***) and knockout (***G***) are similar, with indistinguishable *I–V* relations (***D***, ***H***).

With the goal of evaluating the functional capacities of horizontal cells of *Cx57-VGAT^−/−^* mice, we characterized their intrinsic properties to test for possible differences that could affect feedback. We assessed inward rectifier K^+^ currents, outwardly rectifying K^+^ currents, and inward Ca^2+^ channel currents using whole-cell voltage clamp of isolated horizontal cells identified by their expression of tdTomato. Figure 3 compares whole-cell membrane currents recorded from control, *Cx57-tdTomato-VGAT^+/+^,* and *Cx57-VGAT^−/−^* knockout mice, and they showed no discernible difference. Inward rectifier K channels (Tachibana, 1983) and outwardly rectifying K currents (Feigenspan et al., 2009) were the same in *VGAT^+/+^* and *VGAT^−/−^* mice, as judged by the peak current amplitudes and the kinetics of the voltage-dependent activation. Inward rectifier K^+^ (Kir) currents had a peak magnitude of −354 ± 123 pA at −150 mV in control *VGAT^+/+^* mice (*n* = 9) and an insignificantly different −288 ± 87 pA in *VGAT^−/−^* mice (*p* = 0.67; *n* = 8; Welch’s *t* test^a^). Outwardly rectifying K^+^ (Kv) currents in *VGAT^+/+^* mice had a peak magnitude of 2018 ± 845 pA at +40 mV (*n* = 9), while in *VGAT^−/−^* mice, the peak amplitude was not significantly different at 1765 ± 418 pA (*p* = 0.79; *n* = 9; Welch’s *t* test^b^). Amplitude and kinetics of Ca channel (Cav) currents in horizontal cells (Schubert et al., 2006) carried by Ba^2+^ were indistinguishable between *VGAT^+/+^* and *VGAT^−/−^* mice. For control mice, Ca channel currents had a peak amplitude of −151 ± 42 pA at −10 mV (*n* = 20), with a membrane potential for half activation (*V*_1/2_) of −13.0 ± 6.2 mV, while in *VGAT^−/−^* mice the peak amplitude, occurring at the same membrane potential, was −130 ± 39 pA (*p* = 0.72; *n* = 10; Welch’s *t* test^c^), and the *V*_1/2_ was −17.1 ± 6.6 mV (*p* = 0.66; *n* = 10; Welch’s *t* test^d^), with neither parameter showing a significant difference. These results indicate that the voltage-gated ion channels of horizontal cells from *VGAT^−/−^* mice have normal properties and that the cells should be capable of producing wild-type membrane potential responses to stimulation.

**Figure 4.**
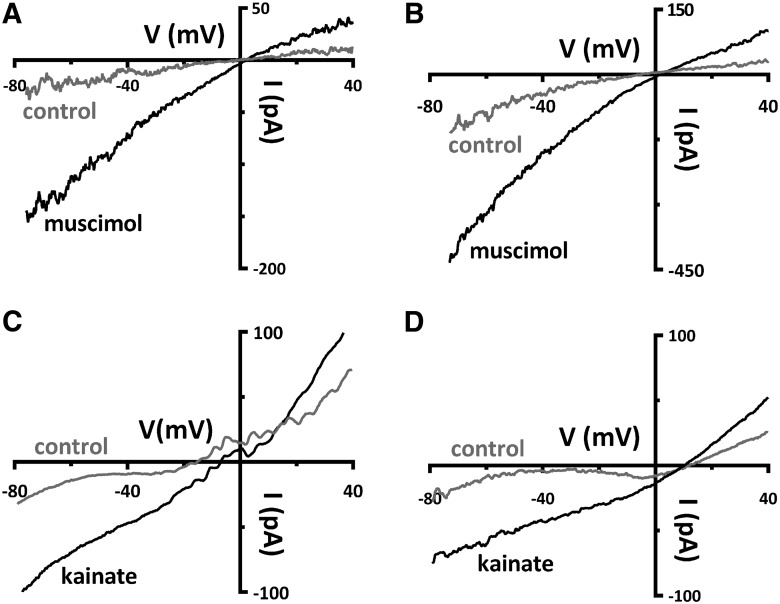
Excitatory and inhibitory ligand-gated currents are present in horizontal cells from *Cx57-VGAT^−/−^* retinae. ***A***, ***B***, Whole-cell voltage-clamp recordings, with CsCl-filled pipettes, of isolated horizontal cells from control *Cx57-tdTomato VGAT^+/+^* (***A***) and knock-out *Cx57-VGAT^−/−^* mice (***B***) show that ionotropic GABA receptors in horizontal cells are unaffected by the deletion of VGAT. Currents in response to ramps (−80 to +40 mV, 240 ms) are shown before (control) and during bath superfusion of 50 μm muscimol, a GABA_A_R agonist. The slope conductance of the current–voltage (*I–V*) relations increased in both in *Cx57-VGAT^+/+^* and *Cx57-VGAT^−/−^* mice during muscimol superfusion (values summarized in text), although the muscimol-induced conductance was larger in the knock out animals. ***C***, ***D***, Glutamate receptor function was tested with 50 μm kainate superfused on *Cx57-VGAT^+/+^* (***C***) and *Cx57-VGAT^−/−^* horizontal cells (***D***). Currents in response to ramps are shown before and during bath superfusion of 50 μm kainate. The increases in slope conductance of the *I–V* relations during kainate superfusion were not significantly different in horizontal cells from *VGAT^+/+^* and *VGAT^−/−^* mice (values summarized in text).

The feedback mechanism recently proposed to operate in the rat retina involves horizontal cell release of GABA onto themselves in an autocrine manner, and our experimental paradigm for testing this relies on the depolarization of the horizontal cells with the iGluR agonist, kainate, and competent GABA_A_ receptors (Liu et al., 2013). Therefore, the functionality of GABA and glutamate receptors was compared in *Cx57-tdTomato-VGAT^+/+^* and *Cx57-VGAT^−/−^* mice to assess possible changes to these horizontal cell neurotransmitter receptors. Application of the GABA_A_ receptor agonist 50 μm muscimol in isolated horizontal cells from both *Cx57-VGAT^+/+^* and *Cx57-VGAT^−/−^* produces slope conductance increases (Fig. 4*A*,*B*). When muscimol (50 μm) was superfused, the normalized conductance increased significantly by 0.55 ± 0.20 nS in *VGAT^+/+^* mice (*p* = 0.03; *n* = 6; Wilcoxon signed rank test^e^). In *VGAT^−/−^* mice, the muscimol-induced increase in conductance was 2.72 ± 0.43 nS (*p* = 0.02; *n* = 3; paired *t* test^f^). In *VGAT^+/+^* mice, five of six cells recovered to 103% ± 41% after washout of muscimol, while in *VGAT^−/−^* mice two of three cells recovered to 100% ± 1% in washout (with one cell in each condition not recorded in washout). The increase in conductance induced by muscimol was larger in the *VGAT^−/−^* mice than in the *VGAT^+/+^* mice (*p* = 0.021; Welch’s *t* test^g^). Glutamate receptor function was tested with 50 μm kainate, a mixed AMPA and kainate receptor agonist, on isolated horizontal cells, and control and *VGAT^−/−^* mice responded with similar increases in slope conductance (Fig. 4*C*,*D*). Superfusion with kainate (50 μm) increased the conductance by 1.49 ± 0.22 nS in *VGAT^+/+^* mice (*p* = 0.007; *n* = 4; paired *t* test^h^), while in *VGAT^−/−^* mice the kainate-induced increase was 1.30 ± 0.39 nS (*p* = 0.03; *n* = 5; paired *t* test^i^). In *VGAT^+/+^* mice, three of four cells recovered after washout (to 110% ± 3%), whereas in *VGAT^−/−^* mice, three of five cells recovered to 112% ± 3% in washout (with one and two cells in each condition, respectively, not recorded in washout). The increase in conductance induced by kainate was not significantly different between the *VGAT^+/+^* and *VGAT^−/−^* groups (*p* = 0.69; Welch’s *t* test^j^). These data show that horizontal cells in *VGAT^−/−^* mice are capable of responding normally to GABA and glutamate receptor agonists.

### Inhibitory feedback to photoreceptors is absent in *VGAT^−/−^* mice

With normal electrophysiological properties established for the *VGAT^−/−^* mice, in particular their responses to the neurotransmitters GABA and glutamate, we next assessed output from horizontal cells using calcium imaging in living retinal slices to test the role of VGAT expression in horizontal cells in feedback onto photoreceptors. This assay has been used in retinae from zebrafish (Vessey et al., 2005), salamander (Cadetti and Thoreson, 2006; Thoreson et al., 2008; Babai and Thoreson, 2009), mouse (Babai and Thoreson, 2009), and rat (Liu et al., 2013), and permits analysis of the inhibitory effects of horizontal cell depolarization on the voltage-gated Ca channels of rod and cone photoreceptors. The assay uses the iGluR agonist kainate and the iGluR antagonist NBQX to depolarize or hyperpolarize horizontal cells, respectively, in conjunction with brief applications of 30 mm potassium that depolarize mouse rods from −54 to –35 mV and rat rods from −53 to −41 mV, and elicit Ca influx into the photoreceptors (Babai and Thoreson, 2009; Liu et al., 2013). Kainate, which has a typical EC_50_ in the range of 120-160 µm at AMPA receptors (Huettner, 1990; Patneau and Mayer, 1991), has been reported to depolarize isolated rat horizontal cells by 22 mV (50 µm; Liu et al., 2013), salamander horizontal cells in retinal slices by 31 mV (1 mm puffs), and isolated turtle horizontal cells by ∼50 mV (20 µm; Hirasawa and Kaneko, 2003), whereas 6,7-dinitroquinoxaline-2,3-dione (DNQX; 100 µm puffs), an iGluR antagonist having an EC_50_ of ∼10 µm (Honoré et al., 1988), hyperpolarizes the cells by 12 mV (Babai and Thoreson, 2009). The iGluR agonists and antagonists have no direct effect on photoreceptors (Thoreson et al., 2008; Babai and Thoreson, 2009; Liu et al., 2013; Kemmler et al., 2014). Kainate increases feedback inhibition of Ca channels in photoreceptors from zebrafish, salamander, mouse, and rat, reducing their depolarization-evoked calcium signals; whereas NBQX or DNQX decreases the feedback inhibition of photoreceptors, increasing their depolarization-evoked calcium signals (Hirasawa and Kaneko, 2003; Vessey et al., 2005; Cadetti and Thoreson, 2006; Thoreson et al., 2008; Babai and Thoreson, 2009; Fahrenfort et al., 2009; Liu et al., 2013).

Pharmacological modulation of horizontal cell membrane potential with NBQX and kainate, which short-circuits the inhibitory feedback loop between horizontal cells and photoreceptors, partly mimics the effects of slow timescale, bright full-field illumination, and full-field darkness, respectively. Our use of fluorescence-based calcium imaging with a confocal microscope, which entails bright stimulation with blue light to drive the fluorescent signals that report calcium levels, limited the use of darkness itself to increase the inhibition of the photoreceptors. It may be fruitful to further study these actions of horizontal cell membrane polarization using more natural stimuli. The controlled depolarization of photoreceptors with 30 mm K^+^ stimulates [Ca^2+^]_i_ increases providing strong signal-to-noise ratios, as shown in the numerous previous studies in which this technique has been used.

**Figure 5.**
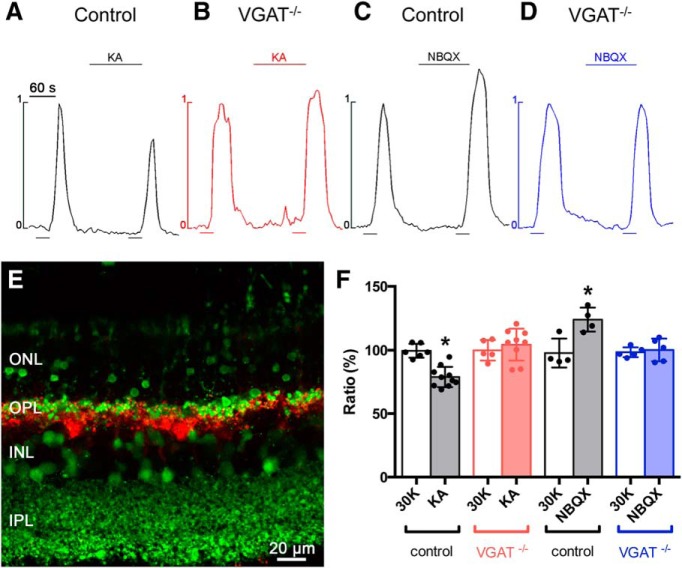
Deletion of VGAT in horizontal cells results in the loss of inhibitory feedback modulation of photoreceptor [Ca^2+^]_i_. Feedback inhibition to photoreceptors was eliminated in retinal slices from *Cx57-VGAT^−/−^* mice. ***A***, In *Cx57-VGAT^+/+^* mice, 50 μm kainate (KA), which depolarizes horizontal cells, inhibited high [K^+^]-evoked calcium signals in photoreceptors (timing shown in bars below traces), suggesting an increase in inhibitory feedback. The high [K^+^] stimulus was always superfused for 30 s, the timing of which is shown by the horizontal bar below the traces. ***C***, 50 μm NBQX, which hyperpolarizes horizontal cells, enhanced calcium signals in photoreceptors, suggesting a decrease in inhibitory feedback. ***B***, ***D***, In *Cx57-VGAT^−/−^* retinal slices, kainate did not increase inhibitory feedback to photoreceptors (***B***), and NBQX did not reduce inhibitory feedback (***D***). Fluorescence traces are shown normalized to the first of each paired response to high [K^+^] application. Calibration: 60 s. ***E***, Confocal image of a *Cx57-tdTomato* retinal slice loaded with fluo-4 (green). The expression of tdTomato (red) identifies the horizontal cells within the slice. Photoreceptor cell bodies are in the outer nuclear layer and their axon terminals are in the OPL. ***F***, Summary of photoreceptor calcium signal amplitudes in retinal slices from *VGAT^+/+^* and *VGAT^−/−^* mice treated with kainate and NBQX. Graph shows normalized mean values ± SDs from multiple retinal slices with one data point (filled circle) per slice, each of which was averaged from 5 to 20 individual photoreceptors from three to five different eyes. ONL, Outer nuclear layer; INL, inner nuclear layer.

In control *Cx57-VGAT^+/+^* mice, 30 mm K^+^ depolarization-induced photoreceptor [Ca^2+^]_i_ signals were reduced by 21% ± 3% (*p* = 0.00003; Welch’s *t* test^k^) when 50 µm kainate was applied and increased by 24% ± 5% (*p* = 0.01; Welch’s *t* test^l^) when 50 µm NBQX was applied (Fig. 5*A*,*C*), confirming previous findings (Vessey et al., 2005; Cadetti and Thoreson, 2006; Thoreson et al., 2008; Babai and Thoreson, 2009; Fahrenfort et al., 2009; Liu et al., 2013). In contrast, kainate and NBQX in *Cx57-VGAT^−/−^* mice failed to produce a change in photoreceptor [Ca^2+^]_i_ (Fig. 5*B*,*D*). In *Cx57-VGAT^−/−^* mice, 50 µm kainate produced an insignificant increase of the photoreceptor calcium signal during the second K^+^ pulse (9% ± 6%; *p* = 0.37; Wilcoxon rank sum test^m^), and 50 µm NBQX produced no change in the calcium signal (0 ± 4%; *p* = 0.70; Welch’s *t* test^n^). Note that kainate and NBQX did not appear to directly affect the photoreceptor [Ca^2+^]_i_, prior to the high-K stimulus. Figure 5*F* summarizes the actions of kainate and NBQX in control and *Cx57-VGAT^−/−^* mice. These findings show that the modulation of photoreceptor Ca channels, the key action underlying horizontal cell feedback inhibition, is eliminated in animals lacking VGAT in their horizontal cells.

## Discussion

The results presented here, achieved by creating a mouse line in which horizontal cells do not express VGAT, the transporter responsible for high-affinity uptake of GABA by synaptic vesicles (McIntire et al. 1997), demonstrate that depletion of VGAT from horizontal cells eliminates inhibitory feedback of photoreceptor [Ca^2+^]_i_ levels. Even though horizontal cells have long been known to send inhibitory signals to photoreceptors and bipolar cells, the mechanisms that underlie this neurotransmission remain unresolved (Thoreson and Mangel, 2012). Pharmacological tests of the physiological role of GABA in the outer retina have provided contradictory results (turtle, Thoreson and Burkhardt, 1990; primate, Verweij et al., 2003; McMahon et al., 2004; Davenport et al., 2008; for review, see Thoreson and Mangel, 2012; rat, Liu et al., 2013; mouse, Pattnaik et al., 2000; Kemmler et al., 2014). In patch clamped turtle cones, GABA-activated Cl^−^ currents were detected only when the GABA receptor-potentiating drug pentobarbital was added (Tatsukawa et al., 2005). In the present experiments, which were performed in mouse retina, no potentiating drugs were added. This suggests that, under the conditions of our recordings, the mouse retina releases enough GABA or expresses sufficient GABA receptors for the slow physiological responses we detected. In cold-blooded vertebrates, a plasma membrane GABA transporter-1 (GAT-1) has been shown to release GABA in an electrogenic, Ca-independent manner (Schwartz, 2002). However, mammalian horizontal cells do not express GATs (Johnson et al., 1996; Guo et al., 2009), suggesting that a different mechanism must be used. It is known that feedback inhibition to photoreceptors depends on horizontal cell membrane potential, but the mechanism by which GABA release might produce this modulation in mammalian retinae has remained unsettled (Thoreson and Mangel, 2012). By generating the *Cx57-iCre* transgenic mouse line, specific genetic manipulation of horizontal cells enabled this study of horizontal cell function in the absence of VGAT on synaptic vesicles.

### Validation of *Cx57-iCre* mouse

Horizontal cell bodies are sparsely distributed, making up only a small percentage of the total number of retinal cells, yet they innervate all photoreceptor terminals (Wässle, 2004). The photoreceptor synapse is complex, consisting of ON bipolar cell dendrites (approximately seven morphological subtypes of cone bipolar cells and one rod bipolar cell) and horizontal cell endings that invaginate the photoreceptor terminal, and approximately five OFF cone bipolar cell types that form synapses at the base of the cone pedicle (Haverkamp et al., 2000; Wässle et al., 2009). The dendrites of type B horizontal cells, the only morphological type in the mouse retina (Peichl and González-Soriano, 1994), innervate cone terminals, whereas the axon terminal system innervates rod terminals (Wässle, 2004). Nevertheless, similar HEPES-sensitive, pH-dependent feedback inhibition of photoreceptor calcium channels appears to occur at rod and cone terminals (Thoreson et al., 2008; Babai and Thoreson, 2009; Thoreson and Mangel, 2012)

The successful development of a *Cx57-iCre* mouse line allowed us to take advantage of Cre-*loxP* technology (Sauer, 1998) and a growing number of Cre-dependent lines and viral vectors to selectively manipulate proteins and cellular pathways in horizontal cells. For example, crosses with Cre-reporter lines, such as *Ai14(RCL-tdT)-D*, produced strong expression of fluorescent proteins in horizontal cells, permitting their targeting for electrophysiological and immunohistochemical analysis. Recently, GluR4 was deleted in mouse horizontal cells using a strategy broadly similar to that used here (Ströh et al., 2013).

The selective loss of VGAT from the horizontal cells did not produce gross alterations in retinal morphology. The light microscopic analysis indicated that the horizontal cell morphology and the OPL appeared unchanged in the *Cx57-iCre* transgenic and the *Cx57-VGAT^−/−^* mouse lines. Similarly, retinal morphology of *Cx57^+/lacZ^* heterozygotes and Cx57^lacZ/lacZ^ nulls was reported to be normal (Shelley et al., 2006; Janssen-Bienhold et al., 2009), as was the synaptic morphology between photoreceptors and horizontal cells (Shelley et al., 2006). Recently, another connexin isoform (Cx50), which couples axon terminals of mouse horizontal cells, was reported (Dorgau et al., 2015).

The functional characterization in this article used hemizygous *Cx57-iCre* mice, meaning that there was a copy of each gene, and Cx57 (and iCre) protein in all horizontal cells. Horizontal cells in heterozygous Cx57^+/−^ mice show reduced tracer coupling, which is reflective of lower Cx57 protein levels (Hombach et al., 2004). However, the homozygous deletion of Cx57 (i.e., a Cx57 knockout), which appeared to eliminate tracer coupling (Hombach et al., 2004), did not disrupt spatial tuning at the ganglion cell level or in behavioral assays (Dedek et al., 2008). Notably, the slow rollback of the photoreceptor light response, considered to arise from feedback, is intact in Cx57 knockout mice, indicating that Cx57 is not essential for the feedback inhibition of photoreceptors (Shelley et al., 2006).

The intrinsic electrophysiological properties of the *Cx57-VGAT^−/−^* horizontal cells appear unchanged from those isolated from wild-type C57BL/6 mice (Schubert et al., 2006; Feigenspan et al., 2009) and *Cx57-iCre^+/−^-tdTomato-VGAT^+/+^* retinae. Significantly, the horizontal cell calcium currents that trigger vesicular release showed amplitudes, kinetics, and current–voltage relations in *Cx57-VGAT^−/−^* mice that were similar to those in *Cx57-VGAT^+/+^* mice. There was also no change in photoreceptor calcium influx following depolarization with control high K^+^ in the two transgenic mouse lines, and the voltage-gated K channels in *Cx57-VGAT^+/+^* and *Cx57-VGAT^−/−^* mice were the same. The response of isolated horizontal cells to the iGluR agonist kainate and to the GABA_A_R agonist muscimol was similar in *Cx57-VGAT^+/+^* and *Cx57-VGAT^−/−^* animals, although the muscimol-induced conductance was larger in the knock out animals. Thus, the loss of feedback inhibition to photoreceptors following VGAT knockout in horizontal cells is not due to altered properties of calcium or potassium channel currents in horizontal cells, nor is it due to a loss of expression of iGluRs or GABA_A_Rs, which are intrinsic to these cells.

### Inhibitory feedback to photoreceptors is absent in the Cx57-*VGAT^−/−^* retina

In wild-type retinae, the depolarization of horizontal cells by kainate or positive current injection produces Ca channel inhibition in photoreceptors due to increased inhibitory feedback from horizontal cells (Thoreson and Mangel, 2012). Likewise, when horizontal cells are hyperpolarized by NBQX or negative current injection, the increase in photoreceptor Ca_i_ indicates that inhibitory feedback is reduced. Feedback inhibition by horizontal cells results in modulation of the photoreceptor calcium current and, consequently, of the release of photoreceptor transmitter (Thoreson and Mangel, 2012).

The manipulation of horizontal cell membrane potential in wild-type retinae with kainate and NBQX generates reproducible modulations of high K^+^-evoked Ca^2+^ signals in photoreceptors in many cold-blooded vertebrates and mammals, including mice, as we show here (Hirasawa and Kaneko, 2003; Vessey et al., 2005; Cadetti and Thoreson, 2006; Thoreson et al., 2008; Babai and Thoreson, 2009; Fahrenfort et al., 2009; Liu et al., 2013). In contrast, in *Cx57-VGAT^−/−^* retinae, kainate depolarization of horizontal cells failed to inhibit high K^+^-evoked Ca^2+^ signals, and NBQX-induced hyperpolarization failed to enhance these signals in photoreceptors, indicating that inhibitory feedback was disrupted because of the inability of horizontal cells to release GABA.

Although the vesicular release of GABA from horizontal cells appears necessary for feedback inhibition onto photoreceptors, there is evidence that the site of action of the GABA is not on photoreceptors directly (Liu et al., 2013). Generally, the evidence for ionotropic GABA receptors on mammalian photoreceptors has been at best equivocal (Thoreson and Mangel, 2012; Kemmler et al., 2014); whereas, ionotropic GABA receptor currents in mammalian horizontal cells (Blanco et al., 1996; Wässle et al., 1998; Feigenspan and Weiler, 2004; Liu et al., 2013) have been demonstrated. Depolarization of horizontal cells was shown to inhibit photoreceptor calcium signals in rat retinal slices (Liu et al., 2013). Since antagonists of GABA_A_Rs blocked this action, it was inferred that an autocrine action mediated by GABA release, acting at GABA_A_Rs on the horizontal cells, was responsible. Since GABA_A_ receptors possess a relatively high permeability for HCO_3_^−^ (Bormann et al., 1987), the resulting flux of HCO_3_^−^ across the horizontal cell membrane during GABA_A_R activation can change the pH in the synaptic cleft between the horizontal cell and photoreceptor axon terminal, where it is known that changes of pH in the cleft modulate the gating of photoreceptor Ca channels (Hirasawa and Kaneko, 2003; Vessey et al., 2005; Cadetti and Thoreson, 2006; Davenport et al., 2008), which leads to altered glutamate release (Barnes et al., 1993). The model unites the vesicular GABA release mechanism in mammals, established in this work, with the more widely accepted pH-sensitive effect (Thoreson and Mangel, 2012).

## Synthesis

The decision was a result of the Reviewing Editor Kirill Martemyanov and the peer reviewers coming together and discussing their recommendations until a consensus was reached. A fact-based synthesis statement explaining their decision and outlining what is needed to prepare a revision is listed below. The following reviewer(s) agreed to reveal their identity: Wallace Thoreson

Both reviewers agreed that the results presented in the manuscript are novel and address highly significant issue in the field: contribution of neurotransmitter GABA released by the retina horizontal cells to feedback inhibition of photoreceptors. The reviewers are also in agreement that the studies are well executed and provide novel insights of fundamental importance. However, before the manuscript can be accepted the following minor issues need to be addressed.

1.Please provide some discussion on the effect of iCre insertion into endogenous locus of Cx57 gene on Cx57 protein expression and its possible role or the absence of thereof in mediating the feedback.

2.Inclusion of reference to Tatsukawa et al., 2005 study is recommended. This study showed that GABA effects on horizontal cell to cone feedback were seen when GABA responses were enhanced by pentobarbital. This suggests that under the conditions of the present study, GABA responses may be enhanced. In addition to citing this work it would be helpful to discuss possible mechanisms for such an elevation (e.g., circadian changes in GABA levels and/or receptors).

3.Some discussion on the limitations of this study related to largely pharmacological nature of manipulations performed rather than naturalistic light stimulation would be helpful to include. More subtle changes in photoreceptor depolarization might have revealed e.g., an effect of NBQX in VGAT-/- animals. For example, in Figure 5D, there seems to be a difference in the integral of the response between the first and second applications of K+. Was this routinely observed? Also, were lower concentrations than 30 mM K+ tested in evoking receptor depolarization? There is a concern that concentration used may generate a saturating response.

4.Pharmacology section would benefit from more attention. A) Please consider parallel sentence construction when describing the results to improve the flow of sentences that compare two conditions. In particular, sentence beginning: 'Normalized, non-leak subtracted slope conductance...' needs help. B) Please report p values with fewer than 4 significant digits. In the instance where p = 0.06 is interpreted as significant, either provide additional recordings to strengthen the statistics or include caveats that the effect is borderline. C) Please clarify the concept of washout. If only one or two cells showed a washout, does that mean the others didn't show a washout? Or did they die before the washout could even be measured?

5.It would be helpful to scrutinize the paper for minor typos and omissions. When doing so, address the following. A) page 1 (abstract), mentions that horizontal cells are capable of releasing 'inhibitory neurotransmitters'. It would be better to simply say 'GABA' unless there is another possibility. B) page 15. 'glutamate [receptor] agonists' (add 'receptor') C) page 15. Reports of effects of kainate and DNQX on various cell responses are uninterpretable without giving the molarity of the drugs. These should, in turn, be given in some context - the EC50 of the drug for a given receptor. D) Figure 5 A-D needs scale bars for both x and y axes.
